# The risks, needs and stages of recovery of a complete forensic patient cohort in an Australian state

**DOI:** 10.1186/s12888-017-1584-8

**Published:** 2018-02-07

**Authors:** Jonathon Adams, Stuart D. M. Thomas, Tobias Mackinnon, Damien Eggleton

**Affiliations:** 1 Custodial Mental Health Service, Justice Health & Forensic Mental Health Network, 1300 Anzac Parade, Malabar, NSW 2036 Australia; 2 Justice and Legal Studies, School of Global, Urban and Social Studies, RMIT University, Building 37, Level 4, Melbourne, VIC 3001 Australia; 3 Justice Health & Forensic Mental Health Network, 1300 Anzac Parade, Malabar, NSW 2036 Australia

**Keywords:** Forensic mental health, Need, Risk, Recovery, Security, DUNDRUM, Canfor, HCR-20

## Abstract

**Background:**

Routine outcome measures are increasingly being mandated across mental health services in Australia and overseas. This requirement includes forensic mental health services, but their utility in such specialist services and the inter-relationships between the measures remain unclear. This study sought to characterise the risks, needs and stages of recovery of an entire cohort of forensic patients in one jurisdiction in Australia.

**Methods:**

Local expert groups, comprising of members of the forensic patient treating teams, were formed to gather information about the status and needs of all forensic patients in the State of New South Wales, Australia. The expert groups provided demographic information and completed three assessment tools concerning the risks, needs and stages of recovery of each forensic patient.

**Results:**

The cohort of 327 forensic patients in NSW appears to be typical of forensic mental health service populations internationally when considering factors such as gender, diagnosis, and index offence. A number of important differences across the three structured tools for forensic patients in different levels of secure service provision are presented. The DUNDRUM Quartet demonstrated interesting findings, particularly in terms of the therapeutic security needs, the treatment completion, and the stages of recovery for the forensic patients in the community. The CANFOR highlighted the level of needs across the forensic patient population, whilst the HCR-20 data showed there was no significant difference in the mean clinical and risk management scores between male forensic patients across levels of security.

**Conclusions:**

To the authors’ knowledge this is the first study of its kind in New South Wales, Australia. We have demonstrated the utility of using a suite of measures to evaluate the risks, needs, and stages of recovery for an entire cohort of forensic patients. The data set helps inform service planning and development, together with providing various avenues for future research.

## Background

The assessment of a patient’s needs has long been recognised as an essential component of health service planning and delivery [1]. The assessment of need is a term used to describe the array of requirements for patients to optimise their recovery, and encompasses a diverse array of biological, psychological, social, cultural, and spiritual factors. Various definitions of ‘need’ have been offered in the literature, encompassing issues relating to quality of life, restoring and maintaining suitable levels of independence, and the ability to benefit from health care [2].

As Phelan et al. have discussed [3], several instruments have been developed to assess the needs of patients with mental health-related difficulties. Initially, these instruments adopted purely what was termed the ‘normative approach’, meaning that they were based solely on the expert views of the treating clinician. An example of this is the Medical Research Council’s Needs for Care Assessment [4] developed by a team in London, England, which was designed to identify areas of remediable need. From the early 1990s onwards, however, the importance of a different, subjective, approach to needs assessment has been promoted, with the centrality of service user involvement in their own treatment and care planning being increasingly emphasized [5].

One of the more commonly used needs assessment schedules in general mental health services is the Camberwell Assessment of Need (CAN) [6]. Four core principles underpinned the development of this tool as an outcome measure; these summarise the generally accepted best practice in needs assessment [3]: firstly, although people with mental illness have some specific needs, most of their needs are similar to those not experiencing mental illness; secondly, people experiencing mental illness may have multiple needs; thirdly, needs assessments should be both an integral part of routine clinical practice and a component of service evaluation; and finally, need is a subjective concept; acknowledging that while patient and staff views can differ they can be equally valid in guiding and evaluating treatment plans.

Routine outcome measures are well established and commonplace across mental health services in Australia and overseas. Since 2003 they have been embedded across the Australian public mental health service system as a means of identifying and responding to the needs of mental health service users, with data being routinely collected and reported. The outcome measures that have been mandated have been developed and validated in general mental health service environments, but some authors have queried their general applicability for use in specialist services [7].

### Need and risk in forensic mental health

A more recent body of work has concentrated on outcome measurement in specialized services, including forensic mental health services. These developments have been based on a growing recognition that while the needs of forensic mental health users may be similar to from those in general mental health services, there are also important distinctions. For example, issues such as personality disorder, offending behaviour, substance use, violence risk, security needs, legislative requirements, and political considerations have been found to be more pertinent with forensic populations [1, 8, 9].

In the 1990s in the United Kingdom several needs assessment studies concentrated on security placement needs and were concerned with population-based needs considerations including, for example, how many high-security or long-stay medium-security beds were needed [1]. One of the drivers for this work was the growing acceptance that a significant number of patients were placed in levels of security not commensurate with their level of need, and hence they were not being placed in the least restrictive treatment environment.

Shaw and colleagues [10] recognised the complexity of forensic mental health and developed a specific questionnaire that measured need in a broader sense, across a variety of domains, including security, dependency, treatment and political needs. Based on these need indices, the authors reported that 261 patients (79%) in secure services were placed at a level of security inappropriate to their needs and risk level, with the majority being placed in a security level that was considered too high.

The appreciation that most needs assessment tools in forensic mental health concentrated on security and risk issues, together with the growing understanding that the needs of forensic mental health users do differ from general mental health, led to the development of the CANFOR [2, 11] – a Forensic adaptation of the well-established Camberwell Assessment of Need. The CANFOR has been used extensively for research and clinical purposes to assess met and unmet needs of forensic patients [12] and has been translated into various languages internationally [13].

The largest international needs assessment study of patients in high secure care occurred in 1999/2000 in the United Kingdom, and was commissioned by the British High Security Psychiatric Services Commissioning Board [12, 14, 15]. The findings were significant in that they determined that 40% of the 1256 patients being held in the three high security psychiatric hospitals in England (Broadmoor hospital, Ashworth hospital and Rampton hospital) were suitable for transfer to services with lower levels of security. Based upon the profiles of need of the patients assessed by the Responsible Medical Officer as requiring different levels of security, a number of similarities and differences were noted in their need profiles that, it was argued, could be used to plan service provision.

Studies elsewhere have since reported similar findings with respect to service needs planning, indicating that, based on a thorough assessment of their risk and needs, a substantial proportion of patients were being held in services not considered to be the least restrictive option. O’Neill et al. [16] showed that 47% of the long-stay patients (>2 years length of stay, *n* = 43) were inappropriately placed across secure services in Dublin, Ireland, 30% of the long-stay patients could be safely transferred to lower levels of security within 6 months, and 63% could be transferred within 3 years. Further, a study by Jacques and colleagues [17] looking at the long-term care needs of patients in a UK medium secure unit found that of 122 male patients in medium security services 25 (21%) had been admitted at least 5 years before. Two distinct groups were apparent in the cohort: a group with chronic challenging behaviour, treatment-resistant mental illness, and need for a high level of support; and a more able group not needing as much support but with a dependency on the hospital.

One of the important distinctions when considering forensic mental health need is the area of violence risk, offending behaviour, and violence risk management. One of the most widely used violence risk assessment tools is the HCR-20 – Historical-Clinical-Risk Management-20 [18]. Version 2 of the HCR-20 has been subjected to more than 200 empirical evaluations and adopted or evaluated in agencies within 35 countries [19].

This international body of research is now being applied in Australia, together with the implementation of forensic mental health needs assessment and risk assessment tools with a view to assessing their applicability and utility as potential outcome measures. In Victoria, Segal et al. [20] found significant positive correlations between staff ratings on the HoNOS-Secure, CANFOR total needs, and CANFOR met needs scores, but no significant correlation with a brief screening assessment of the risk of violence.

Also in Victoria, Abou-Sinna and Luebbers [21] further emphasized the importance of the relationship between needs and risk, reporting significant positive correlations between staff ratings on the HoNOS-Secure, CANFOR-S, and the HCR-20. The authors also found that only the CANFOR-S uniquely contributed variance to the HCR-20. The authors concluded that while the HoNOS-Secure assessed similar underlying domains of need to the CANFOR-S, it did not incorporate a broad range of criminogenic aspects that are related to general reoffending for these individuals.

### Recovery in forensic mental health

Together with the importance of evaluating a broad range of needs in mental health, there has also been an acceptance over recent years of the concept of recovery. In 2013, recovery principles became the guiding principle for mental health policy in Australia, with the launch of the ‘National framework for recovery-oriented mental health services’ [22]. While there is no universally recognised definition or description of recovery, in the National framework recovery is defined as ‘being able to create and live a meaningful and contributing life in a community of choice with or without the presence of mental health issues’. Shrank and Slade [23] have also highlighted the variation in recovery definitions, exploring the concepts of both service-based recovery definitions and user-based recovery definitions.

The applicability of the recovery paradigm is less clearly understood and embraced in sub-specialties of mental health such as forensic mental health, however it has been the topic of some discussion in the literature [24, 25]. For example, Dorkins and Adshead [24] noted the challenges to the recovery agenda in forensic mental health services, including: the values and identity of forensic service users; social exclusion as a community response to trauma and violence; empowerment for those who misuse power and do not respect the choices of others; and hopelessness and the offender identity.

Kennedy et al. [26] have provided the most recent addition to the field of forensic mental health structured professional judgment tools – The DUNDRUM Quartet – which the authors argue provides a validated and transparent means of making decisions about core issues around admission to, transfer between and discharge from forensic mental health services. The DUNDRUM Quartet has been used to evaluate forensic patient discharge and movements between levels of security, with the authors demonstrating the tool’s validity in forensic mental health services [27–31].

As a consequence of there being little consensus regarding which outcome measures may be most appropriate for use in forensic mental health contexts [32], a review [9] was established and funded by the Forensic Mental Health Information Development Expert Advisory Panel – a transnational (Australia and New Zealand) committee established to provide direction for the future development of forensic mental health services. Of 19 possible tools identified, Shinkfield and Ogloff concluded that six broadly met requirements to be considered as feasible for use as an outcome measure for forensic mental health services. These were: DUNDRUM, HoNOS-secure, Short Term Assessment of Risk & Treatability (START), CANFOR, Illness Management & Recovery Scales (IMR) and Mental Health Recovery Measure (MHRM). The authors recommended that because none of the tools by themselves met all of the identified requirements, that services consider using a ‘suite’ of measures.

Despite this review, the use of specialised outcome measures in forensic mental health services remains in its relative infancy in Australia; to date there have been no Australian-based studies evaluating the needs, risks, and stages of recovery of an entire forensic patient population. An understanding of this information is the critical first step in decision making about service provision and planning at a systemic level. The focus of this study was to consider the need for therapeutic security, risks, and stages of recovery of all forensic patients across the State of New South Wales (NSW) in Australia.

## Methods

### Study setting

Forensic patients in NSW are defined by the Mental Health (Forensic Provisions) Act 1990 (NSW). The majority of the forensic patient cohort is made up of two groups: (1) those found not guilty by reason of mental illness (pursuant to Section 38 of the Mental Health (Forensic Provisions) Act 1990 (NSW)); and (2) those with a ‘limiting term’ (whereby the person was found unfit to stand trial, at a special hearing the offence was found to have been committed on the limited evidence available, and a ‘limiting term’, analogous to a sentence, was nominated by the court: Section 23(1)(b) of the Mental Health (Forensic Provisions) Act 1990 (NSW)).

Organisationally in NSW, forensic patients are placed across a range of community, inpatient, and correctional settings, under the governance of differing Local Health Districts (LHDs). While the hospitals are often referred to according to their level of therapeutic security, there are no current agreed standards for each level of security across the State. The Justice Health and the Forensic Mental Health Network (JH&FMHN) oversees the care of the largest cohort of forensic patients, those in the ‘high security’ psychiatric unit and the correctional centres. There is only one ‘high security’ unit in NSW. There are three ‘medium security’ units and two ‘low security’ units, which come under the governance of five different LHDs. There are two ‘open security’ units that are under the same LHDs as two of the ‘medium security’ units. Forensic patients placed in the community are managed by their geographical LHD.

Like many jurisdictions, the placement of forensic patients in NSW is dynamic. For example, patients are admitted to hospitals as necessary, Courts create new forensic patients regularly, and some forensic patients are unconditionally released and are no longer classified a forensic patient. Both the JH&FMHN and the Mental Health Review Tribunal hold databases of the forensic patients in NSW, however, neither of these are live or updated in real time. This created a logistical challenge in terms of identifying a total cohort of forensic patients for this study. To address this, all forensic patients listed in the prison, high security unit, medium security units, low security units, open security units, and community at the time of assessment were eligible for inclusion.

Assessments were completed between December 2014 and July 2015. For reasons of practicality and resourcing a single ‘census date’ was not used.

### Study design

#### Expert group formation

Expert groups were formed to gather information about the needs of the forensic patients. Organisationally, it was anticipated that a range of disciplines from the multidisciplinary team (medical, nursing and allied health specialties) would ideally form the expert group in order to draw on the differing expertise they bring to the assessment of forensic patient needs and assessment of therapeutic security characteristics. This approach is consistent with the work of Shaw et al. [10], and endorsed by Thomas et al. [12] and Pierzchniak et al. [33] as a robust method of assessing forensic mental health need.

Procedurally, the lead clinician at each of the sites was asked to suggest key staff to form an expert group. This individual determined the minimum number of clinicians in their expert group, ensuring adequate coverage of the topics to be addressed during the course of completing the assessment tools. Most commonly the resultant expert group comprised the treating psychiatrist, a member of nursing staff who had a good working knowledge of the patient, and a member of the allied health team. The Community Forensic Mental Health Service (CFMHS) elected only one clinician, who had the best knowledge of the forensic patient, to complete the assessment; in the vast majority of cases this was a psychiatrist. The correctional centre expert group composition varied according to site.

Procedurally, the expert group was guided through each assessment tool by a researcher (JA, TM, DE), rating each item via consensus opinion for each forensic patient in turn. Expert groups referred to the patient medical record, where necessary, to guide their ratings.

The forensic patients considered in the study were not asked to provide consent, for two main reasons. Firstly, no contact with forensic patients was required during this study. Secondly, only members of the expert group had access to the patient medical records, which they routinely had access to for the purpose of fulfilling their primary role. The reviewing Human Research and Ethics Committees approved this approach.

#### Inclusion & Exclusion Criteria

The eligible sample included all adult (above the age of 18 years) male and female forensic patients with a primary diagnosis of major mental illness. Forensic patients found unfit to stand trial (pre-trial) and those with a primary diagnosis of intellectual disability and no major mental illness were excluded. The reason for their exclusion is that these cohorts of patients have significantly different treatment and pathway needs, and the assessment tools have not been specifically validated for this cohort, making them potentially unsuitable. Three of the prison-based forensic patients were excluded from the sample due to an inability to access the treating clinician in the data collection timeframe.

#### Demographic information

Standard details pertaining to gender; date of birth; country of birth; Aboriginal or Torres Strait Islander status; date of admission; legal status (NGMI or Limiting Term); and date of the index offence were recorded.

Index offence was classified according to the Australian and New Zealand Standard Offence Classification (ANZSOC, 3rd Edition) [34], including 16 offence divisions that provide a broad overall picture of offence type. These can be grouped in to broader categories of offences: Divisions 01 to 06 involve offences committed against a person; Divisions 06 to 09 inclusive and Division 12 include offences that generally relate to property; and Divisions 10 and 11, and 13 to 16 inclusive, are offences against organisations, government and the community in general [34].

The expert group provided all psychiatric diagnoses for each forensic patient; general medical conditions were not recorded. The presence of personality disorders was recorded as a dichotomous yes/no variable. The presence or absence of a secondary diagnosed intellectual disability was recorded, and the expert groups could also nominate if an intellectual disability was ‘maybe’ present if this was considered clinically relevant.

For the forensic patients placed in in-patient units the length of stay was calculated as the time from the date of admission to the unit to the date of our assessment. For the forensic patients in the community the length of stay was calculated as the time from the date of discharge in to the community to the date of our assessment.

#### Assessment tools

A range of forensic patient needs were assessed using validated instruments from the perspective of the staff members on the unit who formed the expert group. As per recommendations from Shinkfield and Ogloff [9] a suite of tools were used: the DUNDRUM Quartet [26] – Dangerousness, Understanding, Recovery and Urgency Manual; the CANFOR-S [2] – Forensic version of the Camberwell Assessment of Need (Short version); and the HCR-20 [18] – Historical-Clinical-Risk Management-20 Version 3.

##### *DUNDRUM Quartet* [26] *– Dangerousness, Understanding, Recovery and Urgency Manual*

This manual describes a suite of four structured professional judgement instruments. The instruments focus on: triage security items; triage urgency items; programme completion items; and recovery items. Only three of the four structured professional judgement instruments were utilised however (the triage security items, programme completion items, and recovery items), and the triage urgency items were omitted because they were not relevant to this study. Each item is subdivided in to five, rated from 0 through to 4, which broadly map on to a level for security, with 4 being the highest level of security. Various studies have demonstrated the tool’s validity in forensic mental health services in Ireland [27–31], Freestone et al. validated the DUNDRUM Quartet in a UK forensic service [35], and most recently Eckert et al. demonstrated its utility in Netherlands [36]. The triage security items (DUNDRUM-1, D1) contain 11 items in total, two of which concern self-harm and suicide. Previous studies have demonstrated that the two items concerning self-harm and suicide are poor discriminants [30, 35]. Consequently, in this study the mean item score for D1 excluding the self-harm and suicide related items has been used. This is consistent with the work of Davoren et al. [37]and Eckert et al. [36].

##### *CANFOR-S* [2] *– Forensic version of the Camberwell Assessment of Need (Short version)*

The CANFOR is a widely used individual needs assessment scale designed to identify the needs of people with mental health problems who are in contact with forensic services. It assesses needs in 25 areas of life and covers a broad range of health, social, clinical and functional domains. Each area of need is rated as: no problem – patient has no difficulties in this area and they are not receiving any help in this area; met need – patient has difficulties in this area but is receiving effective help; and unmet need – patient has difficulties in this area and is not receiving help or the help is not effective. Certain items can be rated not applicable. The CANFOR tool has been found to be valid and reliable [11] and has been used in previous needs assessment studies in forensic mental health settings.

##### *HCR-20* [18] *– Historical-Clinical-Risk Management-20 Version 3*

The HCR-20 is a Structured Professional Judgment (SPJ) violence risk assessment instrument. It contains 20 risk factors that span its three subscales: 10 Historical (past) variables; 5 Clinical (present) variables; and 5 Risk (future context) management factors. Each risk factor is rated according to whether it is present, possibly or partially present, or absent. In the HCR-20 V3 these ratings are not scored, but for the purpose of this study: present was scored 2; possibly or partially present was scored 1; and absent was scored 0. It has been demonstrated to be reliable and valid in forensic patient populations [18]. Findings support the concurrent validity and interrater reliability of the Version 3 of the HCR-20 [38]. Its utility in assessing violence risk potential is robust with strong associations with violence (AUCs = .76–.80) [39].

#### Data analysis

Data were entered, coded, managed and analysed using the Statistical Package for the Social Sciences (SPSS v.23.0.0, 2015). Categorical data were crosstabulated and compared using Chi-Squared Tests of Association; where cell numbers fell below *n* = 5 Fisher’s Exact Test statistic was reported instead. Continuous data were first plotted to check its distribution, considering indices of skewness and kurtosis and plotting Q-Q plots. Where parametric assumptions were met, independent t-tests and ANOVAs were computed, the latter utilising Tukey’s HSD post-hoc test to elucidate where group differences occurred. The same analytic decision-making processes applied for correlational analyses, with the male sample scores on CANFOR-S, HCR-20 and DUNDRUM being compared using Pearson’s correlations, while the female scores were correlated using Spearman’s correlations due to deviations from normality with three of the variables and the smaller sample size.

## Results

### Descriptive characteristics of the sample

The complete sample comprised 327 forensic patients. The vast majority were male (89.6%). Male forensic patients were older than female patients at the time of our assessment (mean 45.2 years v 42.9 years), but not significantly so (*t* = 1.079, *p* = 0.281).

In terms of their placement at the time of assessment, approximately one third were placed in high security, one third were in secure hospital settings or prison, and one third were in the community (see Table 1).

**Table 1:** Forensic Patient Placement by Gender

Placement	Gender		Total
	Male (% Within gender)	Female (% Within gender)	
Prison	25 (8.5%)	0 (0%)	25 (7.6%)
High Security	101 (34.5%)	8 (23.5%)	109 (33.3%)
Medium Security	45 (15.4%)	13 (38.2%)	58 (17.7%)
Low Security	8 (2.7%)	1 (2.9%)	9 (2.8%)
Open Security	22 (7.5%)	3 (8.8%)	25 (7.6%)
Community	92 (31.4%)	9 (26.5%)	101 (30.9%)
Total	293	34	327

Thirty-five (10.7%) of the forensic patients had identified as Aboriginal or Torres Strait Islander (ATSI), with significantly more females than males (20.6% v 9.6%, χ^2^ = 3.879, *p* = 0.049). Comparing their country of birth, approximately three quarters of the forensic patients were born in Australia, with no significant differences by gender (Male = 73.6%, Female = 73.5%, *p* = 0.981).

Regarding their forensic patient status, the overwhelming majority (98.2%) were found not guilty by reason of mental illness (NGMI), compared to 6 (1.8%) forensic patients with a limiting term: 4 (1.2%) in the high secure unit, and 2 (0.6%) in the prison setting.

With respect to the index offence, the most serious index offence (according to the Australian and New Zealand Standard Offence Classification, ANZSOC) and the first offence (if there was more than one offence over time) were considered. Male forensic patients were slightly younger, 34.7 years old (SD 11.04), than the female forensic patients, 35.3 years old (SD 8.96), at the time of the first offence but not significantly so (*t* = 0.271, *p* = 0.787). The majority of the offences involved offences committed against a person (*n* = 305), these included: homicide and related offences; acts intended to cause injury; sexual assault and related offences; dangerous or negligent acts endangering persons; abduction, harassment and other offences against the person; and robbery, extortion and related offences [34]. While globally there were no significant differences in broad offence categories type by gender, a more nuanced analysis of the 16 ANZSOC categories revealed that a significantly higher proportion of females engaged in homicide and related offences compared to males (χ^2^ = 3.71, *p* = 0.05).

Considering the sample as a whole, the most common primary diagnosis was schizophrenia (*n* = 234, 71.6%). Comparing gender however, revealed that significantly more male forensic patients were diagnosed with schizophrenia (*n* = 217, 74.1% v *n* = 17, 50.0%, χ^2^ = 8.67, *p* = 0.003) and significantly more female forensic patients were diagnosed with schizoaffective disorder (*n* = 12, 35.3% v *n* = 42, 14.3%, χ^2^ = 9.70, *p* = 0.002). The majority of forensic patients had a substance use disorder diagnosis (*n* = 240, 73.4%); moreover there was a significant association between substance use disorder and gender, with more males than females attracting this diagnosis (*n* = 222, 75.8% vs. *n* = 18, 52.9%, χ^2^ = 8.129, *p* = 0.004). Seventeen (5.2%) forensic patients were diagnosed as having, or maybe having, an intellectual disability. Proportionally more female forensic patients were identified as having an intellectual disability, but this did not reach statistical significance (Fisher’s Exact Test *p* = 0.656). One in five (*n* = 67, 20.5%) of the entire sample was diagnosed with a personality disorder, and there were no significant differences between the genders. The most common personality disorder type for both male and female forensic patients was antisocial personality disorder (Male *n* = 44, 15.0%, Female n = 6, 17.6%).

There were no differences in the average length of stay across the sites for males and females (Male = 2.18 years, Female = 2.2 years, *p* = 0.958).

### DUNDRUM quartet results

#### DUNDRUM-1 across the sites

The mean DUNDRUM-1 score of patients in high security was significantly greater than all of the other sites. The mean DUNDRUM-1 score in the prison setting was significantly higher than all sites apart from high security. The mean DUNDRUM-1 score in medium security was significantly greater than the low and community mean scores, but not the open security sites. There was no significant difference between the low and open, and low and community security mean scores, but the mean DUNDRUM-1 score in open security was significantly higher than the mean score for community forensic patients (see Table 2).

**Table 2:** DUNDRUM Results Across the Sites by Gender

Level of Security	DUNDRUM - Male Forensic Patients						DUNDRUM - Female Forensic Patients					
	DUNDRUM-1		DUNDRUM-3		DUNDRUM-4		DUNDRUM-1		DUNDRUM-3		DUNDRUM-4	
	Triage Security No Suicide		Programme Completion		Recovery		Triage Security No Suicide		Programme Completion		Recovery	
	Mean	SD	Mean	SD	Mean	SD	Mean	SD	Mean	SD	Mean	SD
Prison	2.53	0.83	2.79	0.68	2.48	0.70						
High Security	3.04	0.50	2.81	0.77	2.90	0.70	3.26	0.27	3.71	0.24	3.69	0.31
Medium Security	2.10	0.46	1.89	0.80	1.70	0.64	2.15	0.39	1.85	0.76	1.88	0.55
Low Security	1.51	0.45	1.70	1.29	1.52	1.04						
Open Security	1.74	0.48	1.34	0.60	1.45	0.52						
Community	1.29	0.40	1.50	0.94	1.25	0.71	1.31	0.47	1.68	1.08	1.30	0.79

Note there were no female forensic patients in prison, low security or open security settings

For the female forensic patients, there were significant differences between all three levels of security (high, medium and community) for the mean DUNDRUM-1 scores. The mean scores for the females were higher in high security than medium security, and higher in medium security than in community services.

Cronbach’s alpha for the DUNDRUM-1 ratings was 0.843.

#### DUNDRUM-3 across the sites

For male forensic patients, there was no significant difference in mean DUNDRUM-3 scores between the prison and high security placed forensic patients, however both cohorts scored significantly higher (i.e., less programmes completed) than all other sites. There was no significant difference in mean DUNDRUM-3 scores between medium security, low security, open security, and community placed forensic patients, although there was a pattern of improving programme completion (as evidenced by decreasing mean scores as security levels decreased).

Considering the female forensic patients, the mean DUNDRUM-3 score for forensic patients was significantly higher in high security compared to medium security and community. There was no significant difference in programme completion between females in medium security and those in the community.

Cronbach’s alpha for the DUNDRUM-3 ratings was 0.897.

#### DUNDRUM-4 across the sites

There was no significant difference in the mean DUNDRUM-4 recovery scores between the prison and high security placed male forensic patients, however both cohorts were rated as having recovered significantly less (i.e. their mean DUNDRUM-4 score was higher) than all other sites. There was no significant difference in the mean DUNDRUM-4 scores between medium, low and open security, and no significant difference between the low, open and community, though community forensic patients were rates as being significantly more recovered than medium security forensic patients.

Similar to the DUNDRUM-1 scores, the female forensic patients mean DUNDRUM -4 scores varied significantly across all three levels of security (high, medium and community), with the mean score reducing between high security and medium security, and medium security and community.

Cronbach’s alpha for the DUNDRUM-4 ratings was 0.889.

### CANFOR results

For the males, there was no significant difference in the mean total number of needs between high security, medium security, low security, open security, and the community. However, the male forensic patients in prison settings were rated as having significantly less needs than those in high security, medium security, and the community. The male forensic patients in high security had significantly more unmet needs than those in prison, medium security, open security, and the community, but there was no significant difference compared to the low security cohort (see Table 3).

**Table 3:** CANFOR Results Across the Sites by Gender

Level of Security	CANFOR - Male Forensic Patients						CANFOR - Female Forensic Patients					
	Total Needs		Met Needs		Unmet Needs		Total Needs		Met Needs		Unmet Needs	
	Mean	SD	Mean	SD	Mean	SD	Mean	SD	Mean	SD	Mean	SD
Prison	11.28	3.11	8.84	3.25	2.44	1.73						
High Security	15.29	2.66	10.70	3.17	4.58	2.70	18.75	2.49	15.00	3.78	3.75	1.75
Medium Security	14.56	2.87	12.56	2.77	2.00	1.38	14.69	3.40	13.08	3.15	1.62	1.56
Low Security	13.50	3.63	10.75	2.61	2.75	1.91						
Open Security	13.23	3.01	11.32	2.55	1.91	0.92						
Community	14.95	4.42	12.41	4.22	2.53	2.53	12.44	4.36	9.89	4.65	2.56	2.30

Note, there were no females in prison, low security or open security services

Female forensic patients in high security had significantly more needs than those in medium security and the community. The female forensic patients in high security had significantly more unmet needs than those in medium security, but there was no significant difference compared to the community based female forensic patients.

Cronbach’s alpha for the CANFOR ratings was 0.504.

### HCR-20 V3 results

The male forensic patients in the community had significantly lower H-scores than those in high and medium security, but otherwise there were no significant differences across levels of security. Male forensic patients in high security had significantly higher C-scores than patients in prison, medium security, open security, and the community, but there was no significant difference compared to the low security cohort. There was no significant difference in the mean C-scores between male forensic patients in prison, medium security, low security, open security, and the community. There were no significant differences across any of the sites with respect to R scores. For the mean Total-scores, male forensic patients in high security had significantly higher mean Total-scores than patients in prison, medium security, open security, and the community, but there was no significant difference compared to the low security cohort. There was no significant difference in the mean Total-scores between male forensic patients in prison, medium security, low security, open security, and the community (see Table 4).

**Table 4:** HCR-20 V3 Results Across the Sites by Gender

Level of Security	HCR-20 V3 - Male Forensic Patients								HCR-20 V3 - Female Forensic Patients							
	H		C		R		Total		H		C		R		Total	
	Mean	SD	Mean	SD	Mean	SD	Mean	SD	Mean	SD	Mean	SD	Mean	SD	Mean	SD
Prison	13.16	3.82	3.24	2.85	3.20	1.96	19.60	6.70								
High Security	14.93	3.09	5.87	2.72	3.81	2.28	24.61	6.48	18.00	3.02	8.25	1.67	8.63	1.41	34.88	4.29
Medium Security	14.49	3.38	3.49	2.42	3.22	2.12	21.20	5.81	13.92	3.86	4.23	1.74	3.92	1.93	22.08	5.51
Low Security	14.38	2.07	3.50	3.55	2.38	2.33	20.25	4.74								
Open Security	13.77	3.93	2.73	2.03	2.95	1.40	19.45	5.04								
Community	12.16	3.22	2.76	1.97	3.48	2.34	18.40	5.72	11.33	3.57	2.22	1.99	3.11	2.03	16.67	6.12

Note, there were no female forensic patients in prison, low security or open security settings

Female forensic patients in high security had a significantly higher mean H-score than those in medium security and the community, but there was no significant difference between medium security and the community. There was a significant difference across all sites for the mean C-scores. The mean R-scores showed the same pattern as the mean H-scores, with the female forensic patients in high security having a significantly higher mean R-score than those in medium security and the community, but there was no significant difference between medium security and the community. There was a significant difference across all sites for the mean Total-scores.

Cronbach’s alpha for the HCR scores was calculated: H-scale - 0.656; C-scale - 0.834, R-scale - 0.741.

### Correlations between three outcome measures

For the male forensic patients, the DUNDRUM and all its subscales were significantly positively correlated with the HCR-20 and all its subscales, CANFOR Total Needs, and CANFOR Unmet Needs (apart from CANFOR Total Needs and DUNDRUM-1 which were not significantly correlated). The HCR-20 and all its subscales were significantly positively correlated with the CANFOR Total Needs and CANFOR Unmet Needs (see Table 5).

**Table 5:**
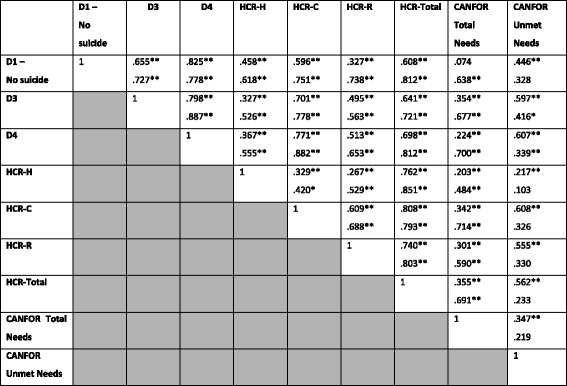
Correlations between DUNDRUM, CANFOR, and HCR-20 Scales and Sub-Scales

^a^Correlation is significant at the 0.01 level (2-tailed) | ^b^Correlation is significant at the 0.05 level (2-tailed)

Male Forensic Patients – Black | Female Forensic Patients – Red

Regarding the female forensic patients a more mixed pattern was evident. The main point of difference was with the CANFOR Unmet Needs, which was not correlated with DUNDRUM-1 or the HCR-20 total score or its subscales.

## Discussion

This study characterised the risks, needs and stages of recovery of an entire cohort of forensic patients across the State of New South Wales in Australia. Broadly speaking the cohort of forensic patients in NSW appears to be typical of forensic mental health service populations internationally when considering factors such as gender, diagnosis, and index offence [14, 31]. The magnitude and direction of the correlations between the three assessment tools considered were broadly consistent with those reported previously by O’Dywer et al. [31]. Additionally, we found that the DUNDRUM sub-scales and the HCR sub-scales have adequate internal consistency, i.e. acceptable Cronbach’s alpha values from 0.70 to 0.95. The lower Cronbach’s alpha for the CANFOR is likely attributable to the broader and more heterogeneous constructs being considered in the needs assessment tool [40].

The results presented here indicate a number of important differences for forensic patients in different levels of secure service provision. Key findings are discussed in turn, followed by service level implications and future research directions.

### DUNDRUM quartet

Generally for both males and females lower mean DUNDRUM-1 scores (a measure of therapeutic security need) were associated with placement in lower levels of therapeutic security. Whilst initially this might be expected, it is interesting to consider that the majority of forensic patients transitioned through the levels of security, starting in high security. An explanation for this might lie in the DUNDRUM-1 tool containing dynamic items, which change as the patient recovers. Alternatively, there might be additional explanatory patient, assessment or system related factors. Since it has been demonstrated that the DUNDRUM-1 items predict length of stay [37], it is to be expected that low-scoring patients will accumulate in lower secure units, and higher-scoring patients will accumulate in higher security, likely taking longer to move on. It may also be that some low-scoring patients are inappropriately admitted to high security when they should have been admitted straight to lower secure places. High-scoring patients are typically characterized by specialist forensic need and complex needs, hence difficulty accessing appropriate treatment programmes may delay their progress from high secure to less secure placements. Lastly, clinicians at lower levels of security may have under-rated due to the length of time since admission and possibly incomplete historical information. All of these factors warrant further empirical scrutiny.

While the DUNDRUM-3 results for programme completion demonstrated a pattern of improvement as the level of security decreased for the male forensic patients, some interesting patterns emerged. Of particular relevance was the finding that community placed forensic patients had not completed significantly more programmes than those in medium security, and also those in low and open security services. A similar pattern was also evident for the male cohort considering their stage of recovery as measured by DUNDRUM-4. These findings are not entirely consistent with other studies. For example, O’Dwyer et al. [31] reported that the DUNDRUM-3 programme completion items distinguished between levels of therapeutic security, while the DUNDRUM-4 recovery items consistently distinguished those given unaccompanied leave outside the hospital and those in the lowest levels of therapeutic security. These discrepancies raise a number of pertinent questions about the configuration and operation of forensic mental health services, including managing the possibility of inappropriately placed forensic patients, the availability of specific forensic mental health programmes (such as treatment programmes addressing problem behaviours and drug and alcohol rehabilitation across all levels of security, but particularly the community based forensic patients), and systemic issues more broadly. That being said, the possibility of this observation relating to a historical or legacy issue should also be considered here. It is possible that for some of those patients, when they transitioned into the community, perhaps a large range of programmes was not available that specifically targeted their forensic mental health needs.

With respect to the female forensic patients the differentiation across the levels of security was clearer for triage security, programme completion and recovery. Although it is not possible to ascertain exactly why the female forensic patients differed to the male cohort in this regard, we would hypothesize that it is indicative of a smaller service structure with more direct and clearly delineated progression determined by a reduction in risk and need.

### CANFOR

A mixed pattern emerged for the male forensic patients both in terms of the number of individual needs they had, and the proportion that were considered unmet. When compared to extant international literature, CANFOR ratings for the total number of needs were consistently higher across all levels of security [14, 20, 21, 31, 41–43]. However, the levels of unmet need were similar to the findings in those studies. The reasons for the former are unclear, but given this study is more recent this possibly reflects a growing recognition of the complexities and diversity of forensic patient need. Perhaps more reassuringly, the level of unmet need was found to be consistent with this prior research, thereby suggesting that while the number of recognised and/or acknowledged needs of forensic patients may have increased, forensic services are, on the whole, continuing to meet these challenges.

Considering the CANFOR results for female forensic patients, the number of total needs and unmet needs reduced as the level of security reduced, similar to the findings for the DUNDRUM. Again, perhaps this is caused by a simpler service structure, with fewer units available across the state catering for female forensic patients, which makes their recovery pathway more clearly stratified. However, caution must be taken as these tools may not fully capture the additionally unique needs of female forensic patients [44, 45].

### HCR-20 V3

As expected given the nature of their offending and the risks inherent in admission to high secure forensic services, the male forensic patient mean H-scores were consistent across all levels of security. The most striking finding from the violence risk data was the lack of significant difference in the mean clinical (C-score, dynamic risk) scores between male forensic patients in prison, medium security, low security, open security, and the community. Additionally, there were no significant differences across any of the sites for the mean future risk management scores. Given the centrality of violence risk management in forensic mental health this is both a surprising and potentially concerning finding. Nevertheless it is consistent with O’Shea et al.’s [46] findings that the HCR-20 dynamic scales are insensitive to change over time. This may well underpin the ongoing concerns regarding the relative lack of community-based supports for this patient cohort who are considered to be at a significant risk of re-offending. This might reflect the historical nature of some of the forensic patients released into the community, perhaps they were released when they were not fully recovered and more unstable compared to the more recently released forensic patients. Also worth considering is whether the HCR-20 V3 had the sensitivity to ascertain the required differences in the NSW context.

The female forensic patients exhibited less of a concerning picture, with a more expected pattern of reducing risk as rated by the HCR-20 and its sub-scales as the level of security dropped. This pattern is similar to the DUNDRUM and CANFOR data for female forensic patients.

### Implications and future research directions

The depth and breadth of data collected in this study gives rise to a number of potential implications, particularly at a whole service level. Ensuring that forensic patients are placed in an environment commensurate with their level of risk, needs, and stage of recovery is a fundamental principle of forensic mental health service provision. This study has revealed potential variations to this principle, particularly in terms of violence risk potential and with the completion of treatment programmes, which will require further careful evaluation in order to better understand. This issue becomes even more relevant as there has been a steady increase in the number of forensic patients in New South Wales over recent years (unpublished data from the Justice Health & Forensic Mental Health Network), which has placed pressure on hospital beds, community placements and patient flow.

A clearer stratification was demonstrated within the smaller female forensic patient cohort, compared to the male cohort, by all three assessment tools. However, there is broad agreement that the needs and recovery pathways for female forensic patients differ to male forensic patients [44, 45, 47]. Hence, the differences observed between the male and female forensic patient cohorts certainly warrant further consideration.

The similarities revealed by the HCR-20 data for the male forensic patients in terms of clinical risk and future risk management are thought provoking, and perhaps unexpected. Assertive risk management, and ultimately the reduction of risk, are of prime importance in forensic mental health care. Although fully understanding the causes for this observation is beyond the scope of this study, the magnitude of risk manifested by male forensic patients, particularly in the community, compels further scrutiny at a service level.

Although not directly measured, a noteworthy positive finding of the present study was the ease of use of the three assessment tools. Participants in the expert groups engaged readily. This might in part explain the readiness of the service providers in the New South Wales Forensic Mental Health Network to consider implementing the assessment tools utilised in this study more formally, which has occurred since this the conclusion of this study. The utilisation of these tools as outcome measures for users of forensic mental health services is supported by the work of Shinkfield and Ogloff [9]. The regular collection and evaluation of this dataset will allow an investigation of the tools’ sensitivity to change, which is a key aspect of demonstrating forensic patient recovery and risk management.

Consideration should be given to the limitations of this study. Arguably the most significant limitation was that all assessment data was gleaned from treating clinicians who comprised the expert groups. Forensic patients and their carers were not surveyed, which is clearly critical clinically. It is positive that there are patient rated versions of the DUNDRUM and the CANFOR available, which opens up avenues for further research and clinical application. Secondly, given the size of the forensic patient cohort in NSW, we were not able to conduct all assessments on a specific census date. Thus, given the dynamic nature of the forensic patient presentations, this might introduce bias in to the results. This could be further explored if the assessment tools used in this study are embedded formally in the Forensic Mental Health Network, and data is routinely collected and evaluated. Thirdly, the exclusion of forensic patients found unfit to stand trial (pre-trial) and those with a primary diagnosis of intellectual disability and no major mental illness should also be acknowledged here. These small but important cohorts of patients require additional nuanced analysis and should be included in broader service development initiatives. Lastly, for ease of comparison, we interpreted the assessment tool data categorically, which potentially excludes relevant case-specific risk, need, and recovery factors. Broadening the scope of further research could include such parameters and enrich the data set.

## Conclusion

By mapping the risks, needs and stages of recovery of an entire cohort of forensic patients in an Australian State we have created a baseline data set that informs service planning and development, together with providing various avenues for future research. The comparability of the NSW cohort to the extant international literature adds weight to the clinical utility of this combination of risk, need and recovery outcome measures across all levels of security in forensic services.
